# Extraction and Verification of Seismic Vibration Metrics via Laser Remote Sensing Utilizing Wavefront Sensors

**DOI:** 10.3390/s26051533

**Published:** 2026-02-28

**Authors:** Donghua Zhou, Quan Luo, Yun Pan, Yiyou Fan, Haoming Chen, Wei Jiang, Jinshan Su

**Affiliations:** 1 Key Laboratory of Vibration Signal Capture and Intelligent Processing, School of Electronic Engineering, Yili Normal University, Yining 835000, China; 20240050197@ylnu.edu.cn (D.Z.); 20230050200@ylnu.edu.cn (Y.P.); 14012@ylnu.edu.cn (Y.F.); 20230050199@ylnu.edu.cn (H.C.); 2 School of Electronic Engineering, Xidian University, Xi’an 710071, China; 25021110995@stu.xidian.edu.cn; 3 Key Laboratory of Intelligent Optical Sensing and Manipulation, College of Engineering and Applied Sciences, Ministry of Education, Nanjing University, Nanjing 210023, China

**Keywords:** laser remote sensing, Shack–Hartmann wavefront sensor, amplitude, vibration velocity, vibrometer

## Abstract

Seismic wave analysis is crucial for identifying subsurface formations and geological hazards. In this study, a seismic wave laser remote sensing system based on a Shack–Hartmann wavefront sensor was established by exploiting its high spatial resolution, array-based detection capability, and independent microlens spot centroid measurement. This method was employed to analyze the correlation characteristics among vibration-related physical variables. Experiments were conducted to assess the quantitative correlation between vibration amplitude and spot centroid shift by the Shack–Hartmann wavefront sensor across a range of 0.06–5.94 mm. Accordingly, based on the measured centroid shift, vibration velocity was derived and validated through comparison with reference vibrometer measurements. In addition, the correlation between vibration amplitude and vibration velocity was systematically analyzed. The experimental results demonstrate strong linear correlation between amplitude and both spot centroid shift and vibration velocity, with coefficients of determination $R^{2}$ exceeding 0.98. The vibration velocity obtained by the proposed system shows strong agreement with vibrometer data, confirming its effectiveness for low-frequency vibration detection. Measurement accuracy can be further improved by reducing noise. These results indicate that the proposed approach provides a promising laser remote sensing solution for seismic wave detection.

## 1. Introduction

Seismic wave exploration is a fundamental technology for elucidating subsurface structures, identifying resource reservoirs [1], and evaluating engineering geological conditions. The essence is efficiently reversing the spatial distribution of subsurface media by analyzing and processing surface observation data [2,3,4]. As the demand for deeper resource exploration, comprehensive geological structure analysis, and geological hazard risk assessment escalates, the need for precision and resolution in subsurface imaging is likewise intensifying [5]. Conventional seismic exploration techniques often depend on closely spaced contact-type geophone arrays for data collection, with imaging quality frequently limited by various causes [2].

In this context, optical remote sensing technology, with its non-invasive and high-resolution capabilities, presents novel opportunities for seismic data exploration. It enhances the precision of seismic waveform analysis and the elucidation of subterranean structures [6,7]. In recent years, wavefront sensors have been implemented in ground target detection, obtaining target information by sensing phase variations in wavefronts [8,9]. Li et al. [10] achieved high-precision acquisition of wavefront data through the use of Shack–Hartmann wavefront sensors. Nonetheless, current methodologies primarily obtain vibration data from individual stations, complicating the analysis of regional vibration fields and indicating potential enhancements in detection efficiency. Luo et al. [11] suggested utilizing the non-interferometric characteristics and arrayed detection capabilities of microlens arrays to concurrently acquire multiple vibration signals without intricate interferometric optical pathways, presenting an innovative method for obtaining high-spatial-resolution wavefield data. The laser Doppler vibration measurement method [12] has proven effective for the long-range detection of subtle vibrations. Nonetheless, it is constrained in delivering high-density spatial gradient data necessary for wavefield reconstruction and full-waveform inversion.

Extensive research has focused on the acquisition and utilization of velocity in vibration data analysis. Virieux et al. [2] achieved medium parameter inversion from seismic recordings by full wavefield modeling; however, this method was not applied to actual observational signals. Liang et al. [13] formulated an empirical forecasting model for blast vibration velocity by integrating data from comparable research. Fu et al. [14] investigated vibration velocity characteristics at specific locations in pile-raft-supported embankments and foundations; Kong [15] employed numerical analysis to predict variations in blast vibration velocity; Shi [16] formulated control standards for peak particle vibration velocity in subterranean pipelines. Nonetheless, this research mostly focuses on vibration analysis within traditional engineering domains, such as railroads and blasting, and fails to effectively include optical vibration signal detection. Additional investigation is required, especially in acquiring vibration parameters from wavefront sensor data.

Currently, there remains an opportunity to extend laser remote sensing techniques to construct quantitative interpretation models and convert observational data into physical parameters suitable for geological analysis. This work utilizes the benefits of wavefront sensors to enhance detection precision andbroaden the applicability of vibration measurement [11]. By capturing the spot centroid shift of the sensor, vibration data can be measured more accurately, allowing the derivation of vibration parameters that are closely aligned with those obtained from conventional vibrometer measurements. Additionally, this approach facilitates the analysis of relationships between vibration amplitude and key derived parameters.

This study focuses on vibration signal acquisition, parameter derivation, and correlation verification based on the Shack–Hartmann wavefront sensor laser remote sensing detection system. Section 1 delineates the fundamental structure of this detecting system and the alterations in the wavefront during oscillation. Section 2 covers the mechanics of laser attenuation during transmission and outlines the theoretical framework used to derive vibration parameters, as well as the impact of temperature and humidity on wavefront sensor performance. Section 3 experimentally validates the linear correlation between vibration amplitude and spot centroid shift under various conditions, compares vibration parameters obtained from centroid shift with vibrometer measurements, and further investigates the relationship between vibration amplitude and the value of vibration velocity.

## 2. Seismic Wave Laser Remote Sensing Detection System

Seismic waves are chiefly categorized into P-waves, S-waves, and surface waves according to their mode of propagation [17,18]. S-waves exclusively travel through solids and mostly induce horizontal ground shaking, rendering them challenging to detect. Surface waves are predominantly interference waves. P-waves, being progressive waves, are the first seismic waves to arrive at the surface of the planet, making them more easily detectable [19]. Thus, observational analysis predominantly concentrates on P-waves [20]. P-waves generate vertical ground motion during an earthquake. This motion modifies the wavefront structure of incoming light, thus incorporating seismic wave attributes into the backscattered laser light [11].

**Figure 1:**
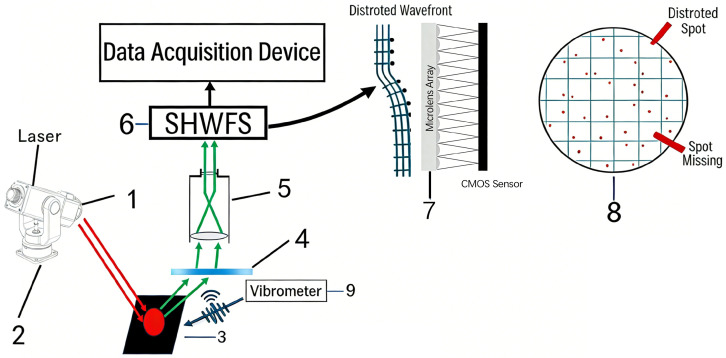
Seismic wave laser remote sensing detection system based on wavefront sensors. 1 Collimating lens; 2 Laser steering device; 3 Target area; 4 Filter; 5 Focusing lens; 6 Shack–Hartmann wavefront sensor; 7 Microlens array; 8 Detection window; 9 Vibrometer.

Figure 1 illustrates the comprehensive architecture of the seismic wave laser remote sensing detection system utilizing wavefront sensors. This system comprises two main components: transmission and signal reception. The transmission end comprises a laser source, a collimating lens (1), and a laser steering device (2). The laser source produces a continuous beam at a wavelength of 635 nm. Upon traversing the collimating lens (1), the beam generates a consistent and stable light projection that illuminates the target area (3) at a predetermined angle and intensity. The receiving apparatus consists of focusing lens assembly (5), a filter (4), and a Shack–Hartmann wavefront sensor (6). The focusing lens (5) acquires reflected and scattered light signals from the target area (3). The filter (4) eliminates extraneous light from the surroundings, hence improving signal integrity. Thereafter, the optical signal proceeds to the Shack–Hartmann wavefront sensor (6) for wavefront analysis. This sensor utilizes a microlens array for wavefront segmentation and detection. Each microlens corresponds to a subaperture, locally concentrating the incident wavefront onto the image plane of the CMOS detector. The microlens array (7) partitions the incident light spot into subapertures, presenting each within its corresponding detection window (8). Simultaneously, the vibration measurement device (9) conducts synchronous vibration monitoring of the target area (3) to obtain real-time vibration data for later comparison analysis.

**Figure 2:**
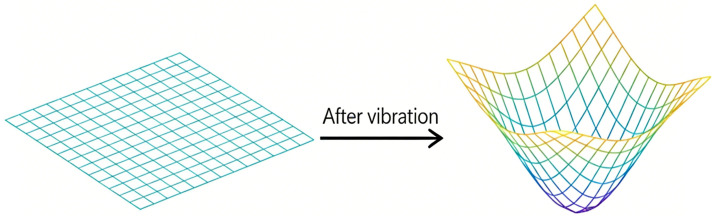
Vibration wavefront.

The system operates based on detecting wavefront variations induced by vibrations, which cause the centroid of the spot to deviate from its reference coordinate, thereby producing a measurable offset. By quantifying and analyzing this offset, vibration characteristics can be effectively extracted. Accordingly, this system is utilized to detect micro-vibrations within the target region. A precise understanding of the relationship between the vibration amplitude of the target and the resulting displacement of the receiving point on the CMOS sensor is essential for accurate measurement. In the absence of vibration, the wavefront remains planar. Upon excitation, the wavefront transitions from planar to concave, as depicted in Figure 2. It should be noted that, for the sake of analytical simplicity, the target surface is assumed to be flat under non-vibrating conditions in order to elucidate the fundamental relationship between the incident and reflected wavefronts. In practice, however, surface irregularities may combine with other non-ideal factors to affect the shape of the wavefront.

**Figure 3:**
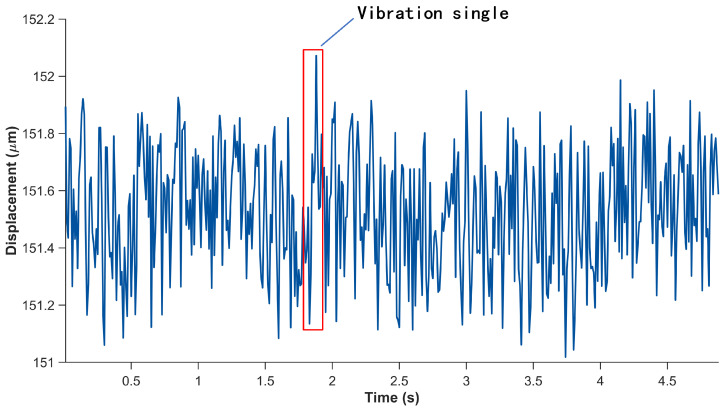
Aliasing noise in response to vibration.

During the measurement process, the vibration signals captured by the system are subject to aliasing noise as well as parasitic signal components arising from unintended or spurious vibrations of the detection apparatus. Figure 3 illustrates the vibrational response of the system and the corresponding noise components observed throughout the measurement procedure.

**Figure 4:**
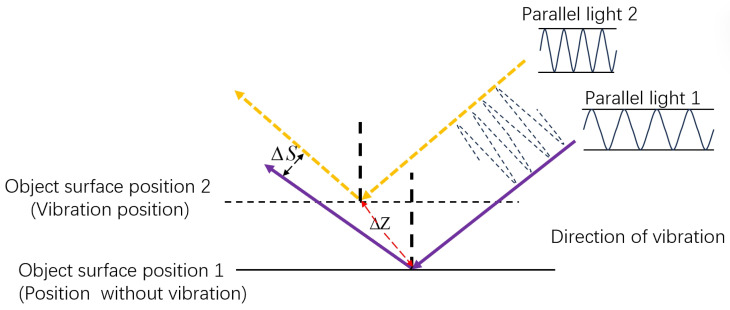
Relationship between vibration amplitude and reflected laser.

Seismic sources produce vibrations that cause longitudinal motion in the medium, which modifies the frequency and phase of the laser echo signal due to P-waves. The source’s continual vibration causes real-time variations in the phase of the reflected laser. The laser wavefront, after reflecting off the ground surface, experiences phase distortion from ground vibrations, thus conveying information on the vibration characteristics [21]. The intensity distribution of the light field captured by each subaperture in the microlens array of the wavefront sensor varies with the incident wavefront. This results in proportional changes in the form and position of the concentrated spot, ultimately appearing as displacements in the spot’s center of mass. Consequently, identifying alterations in the laser wavefront facilitates the collection of seismic wave data produced by terrestrial vibrations. When a target object experiences vibrations of differing strengths, the displacement of the optical spot’s center of mass in the wavefront sensor varies accordingly [22], as depicted in Figure 4.

Recent investigation [23] has demonstrated that longitudinal wave information embedded in the returned signal can be extracted by analyzing variations in the laser wavefront. Experiments reveal that the spot centroid shift ΔS measured by a Shack–Hartmann wavefront sensor varies with the vibration amplitude ΔZ. Based on this phenomenon, ΔS is hypothesized to be proportional to ΔZ, as expressed in Equation (1).

(1) ΔS=kΔZ

In this context, *k* represents a constant.

## 3. Theoretical Framework

### 3.1. Theory and Modeling of Wavefront Sensor Detection

A wavefront sensor is an optical instrument that accurately measures the phase distribution of light waves. The primary function is the continuous capture and analysis of beam wavefront aberrations, facilitating phase calibration for optical systems [24]. In seismic wave laser remote sensing, this technology identifies phase alterations in light waves induced by surface micro-vibrations, facilitating the investigation and inference of the dynamic response characteristics of underlying geological formations [25].

**Figure 5:**
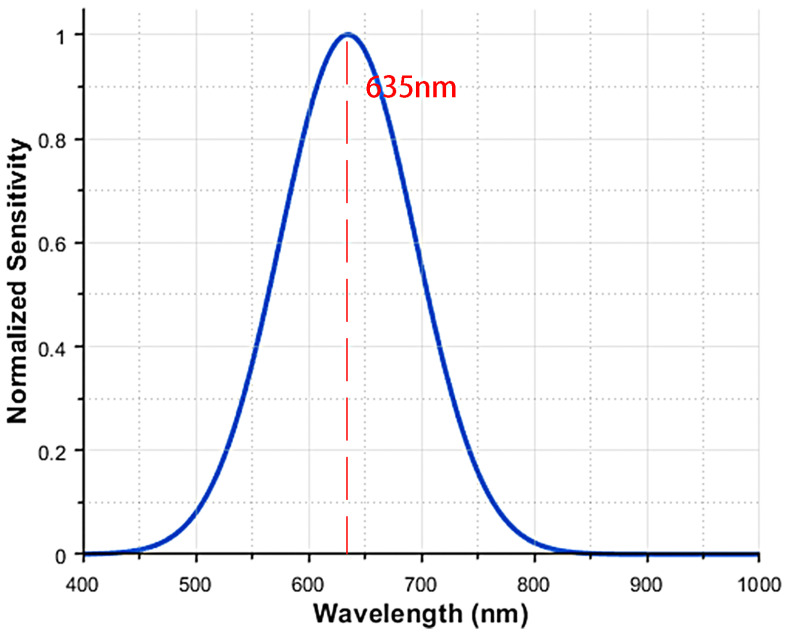
Sensitivity of measurements over various laser wavelengths.

In experiments involving vibration signal detection for laser remote sensing, a Shack–Hartmann wavefront sensor (SHWFS) was employed to characterize laser wavefronts. The sensor exhibited wavelength-dependent responses under practical operating conditions, leading to distinct variation patterns, as illustrated in Figure 5. Note that the sensitivity here represents the normalized system-level vibration measurement response rather than the quantum efficiency of the CMOS detector.

This sensitivity curve model adheres to the classic Gaussian function equation:

(2) $$S\left(\lambda \right)=e^{-\frac{{\left(\lambda -\lambda_{m}\right)}^{2}}{2\sigma^{2}}}$$

Among them, S(λ) denotes sensitivity at wavelength λ; λ represents the wavelength; $\lambda_{m}$ indicates the wavelength at which the associated sensitivity attains its highest value; σ signifies the standard deviation of the Gaussian distribution, regulating the curve’s breadth.

**Figure 6:**
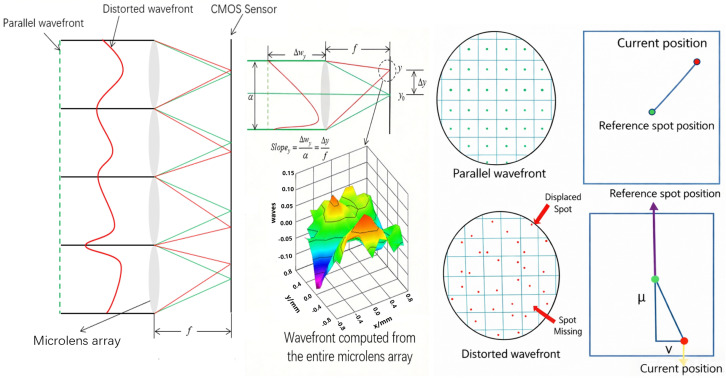
Imaging principle of an individual microlens.

SHWFS functions at elevated velocities with high frame rate sampling proficiency, facilitating swift reactions to subtle laser beam fluctuations and exhibiting considerable benefits in wavefront identification [26]. This sensor comprises two main components: a microlens array and a CMOS sensor [27]. The microlens array consists of several subapertures organized in a systematic arrangement, with each subaperture representing a specific local wavefront region. Each microlens concentrates the laser beam onto the CMOS, creating an array of dots. The CMOS documents the location of each subaperture’s spot and computes its deviation from the reference point. The spots from each subaperture are accurately concentrated at the theoretical focal point of the microlens; this configuration represents the reference spot. If the incoming laser displays phase distortion, the spots from each subaperture diverge from their respective theoretical focal points, resulting in the observed spots [28]. Figure 6 depicts the imaging principle of an individual microlens. The centroid shift of the spot can be determined by comparing the positional offset between the actual measured location and the reference location.

### 3.2. Establishment of Transmission Attenuation Model

**Figure 7:**
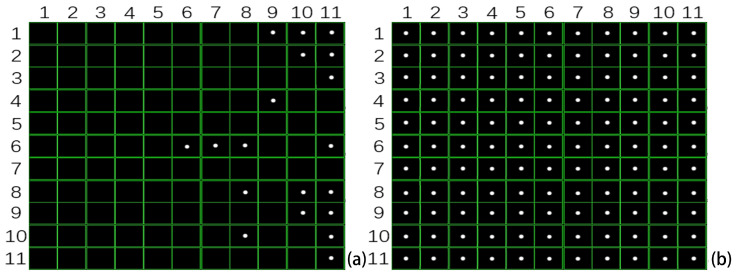
Distribution of spot arrays at varying laser energy levels. (**a**) Inadequate laser energy resulted in sparse spots; (**b**) adequate energy produced a complete and uniform spot array.

When the laser emitter illuminates a target area with constant power, the wavefront sensor receives the reflected and scattered laser signals. Atmospheric transmission attenuation of the laser is influenced by both system characteristics and environmental conditions. If the laser power reaching the wavefront sensor is insufficient, the Wavefront Sensor software (version 18183-D03) will indicate inadequate laser energy. In such cases, only sparse and irregularly distributed local spots are observed on the 11 × 11 microlens array, as shown in Figure 7a. When the laser intensity is sufficient to illuminate the entire array, each microlens focuses the incident beam onto the CMOS target plane, producing a complete and uniformly distributed 11 × 11 spot array, as illustrated in Figure 7b.

By modifying parameters such as transmission distance (L), laser wavelength (λ), and power intensity $\left(P_{0}\right)$, the propagation characteristics of the laser can be altered. The atmospheric structure is a crucial environmental element affecting energy attenuation. From a physical transmission standpoint, the energy attenuation model is categorized into microscopic and macroscopic dimensions: the microscopic level encompasses molecular absorption cross-section σ(λ) and aerosol scattering phase function $\beta \left(\theta \right)$; the macroscopic level pertains to atmospheric optical depth, where the absorption coefficient α(λ) and scattering coefficient β(λ) are influenced by meteorological factors such as pressure gradient ∇p, relative humidity (RH), visibility (VIS), temperature (T), and atmospheric pressure (P). Microscopic analysis offers considerable theoretical precision; however, its computational complexity limits practical application. Thus, field investigations frequently combine microscopic models with macroscopic meteorological surveys, improving feasibility by utilizing the correlation between laser power and detection distance.

The correlation among transmittance T(λ), transmission distance *L*, and attenuation coefficient μ(λ) is articulated as

(3) $$T\left(\lambda \right)=\frac{P_{a}}{P_{0}}=\exp\left(-\mu (\lambda )\cdot L\right)$$

Here, $P_{0}$ signifies laser power prior to attenuation, while $P_{a}$ indicates laser power subsequent to attenuation. Sometimes, the attenuation coefficient is defined as the attenuation value of light signal power over 1 km of atmosphere; denoted as α(λ), it describes the power loss per unit length (1 km). Its relationship with μ(λ) can be expressed as

(4) $$\alpha (\lambda )=-10\mathrm{lg}\frac{P_{a}}{P_{0}}=-10\mathrm{lg}\left[\exp\left(-\mu \left(\lambda \right)\cdot 1\right)\right]=4.343\mu \left(\lambda \right)$$

In the above formula, μ(λ) is expressed in ${\mathrm{km}}^{-1}$, α(λ) in dB/km, and λ represents the wavelength. For specific wavelength lasers, it can also be abbreviated as α and μ.

**Figure 8:**
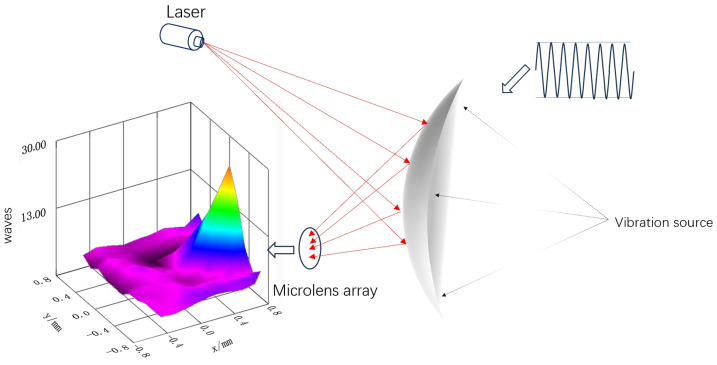
Optimal laser illumination of the target region.

Under optimal conditions, the attenuation coefficient generally remains minimal when the laser lights the target area throughout a specific distance. This idealized process is illustrated in Figure 8.

The correlation among the target point laser power *P*, attenuation coefficient μ, and transmission distance *L* is delineated as follows:

(5) $$P=P_{0}\times e^{\left(-\mu \cdot L\right)}$$

The lighted area of each target point under laser irradiation of a specific power is

(6) $$S=\pi \times {\left(\frac{\Phi }{2}\right)}^{2}$$

Among them, ϕ is the diameter of the laser beam upon arrival at the target location.

(7) $$\Phi =2\times \tan\left(\frac{\theta }{2}\right)\times L$$

Here, θ represents the beam divergence angle, whereas *L* denotes the distance from the emitter to the target area.

**Figure 9:**
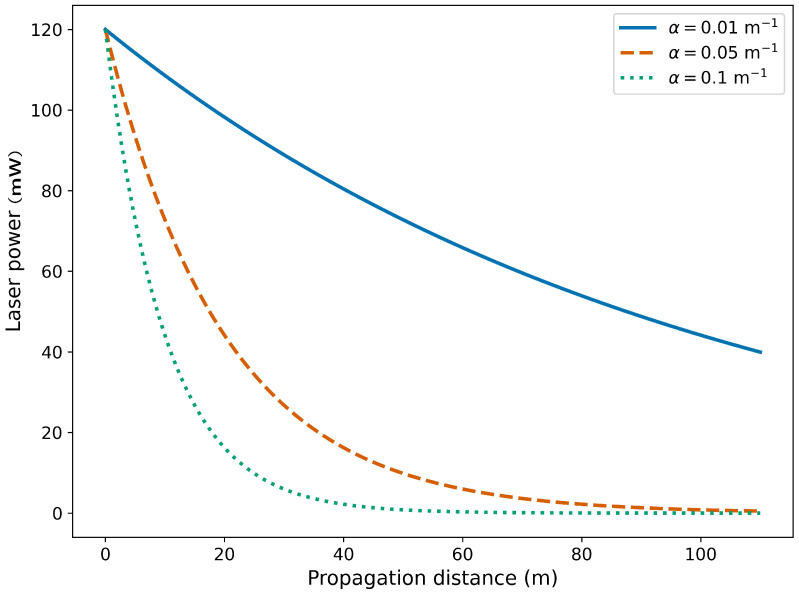
Relationship between laser power and detection distance.

The relationship between laser power and detection distance is illustrated in Figure 9.

### 3.3. Derivation of Vibration Velocity

During actual sampling, slight variations in sampling intervals may arise. Consequently, the effective sampling interval is defined by the average sampling duration to determine the actual sampling rate.

For the initial time series $t\left(l\right)\;\left(l=1,2,\ldots ,N_{\mathrm{raw}}\right)$, the temporal interval sequence between consecutive sampling points is

(8) $$\Delta t_{\mathrm{list}}\left(l\right)=t\left(l+1\right)-t\left(l\right)\;\left(l=1,2,\ldots ,N_{\mathrm{raw}}-1\right)$$

The arithmetic mean of the time difference sequence produces the average sample period:

(9) $$\bar{\Delta t}=\frac{1}{N_{\mathrm{raw}}-1}\sum_{l=1}^{N_{\mathrm{raw}}-1}\Delta t_{\mathrm{list}}\left(l\right)$$

The sampling rate is the inverse of the sampling time; hence, the real sampling rate is

(10) $$F_{s}=\frac{1}{\bar{\Delta t}}$$

Velocity is the initial derivative of displacement in relation to time. The calculation is performed numerically utilizing the forward difference approach, employing the formula:

(11) $$v_{m/s}\left(l\right)=\frac{x_{m}\left(l+1\right)-x_{m}\left(l\right)}{\bar{\Delta t}}\;\left(l=1,2,\ldots ,N_{\mathrm{raw}}-1\right)$$

where $x_{m}$ is the spot centroid shift, measured in m.

To synchronize the number of points in the velocity series with the displacement sequence, NaN values are appended to the beginning, and the unit is transformed into mm/s. Formula (11) undergoes the following transformation:

(12) $$v_{\mathrm{mm}/s}\left(l\right)=\left\{\begin{array}{cc} \mathrm{NaN}, & l=1\\ v_{m/s}\left(l-1\right)\times 10^{3}, & l=2,3,\ldots ,N_{\mathrm{raw}} \end{array}\right.$$

### 3.4. Influence of Temperature and Humidity on the Performance of Wavefront Sensor

**Figure 10:**
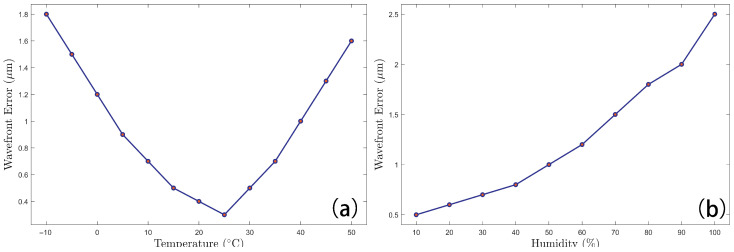
Effects of temperature and humidity on Wavefront Sensor. (**a**) Temperature; (**b**) Humidity.

The measurement accuracy of the 635 nm laser-based system is substantially influenced by environmental conditions. High humidity is characterized to exacerbate laser scattering and absorption. This process induces wavefront distortion, consequently degrading the measurement precision. Furthermore, temperature variations exert a significant impact on system performance: low temperatures retard the sensor’s response time, whereas high temperatures lead to device performance degradation. All of these effects ultimately compromise overall system sensitivity. These combined effects are depicted in Figure 10. It should be noted that the wavefront error variation observed under temperature and humidity changes is not solely attributed to atmospheric effects, but it also includes contributions from thermal drift of the microlens array, detector response variation, and calibration mismatch, which are not actively compensated in the current system.

## 4. Experimental Results and Analysis of Laser Spot Scanning for Vibration Signal Detection

### 4.1. Experimental Environment

Laser transmission in the atmosphere is vulnerable to numerous environmental perturbations. Aerosol particles generate laser scattering and signal attenuation, whereas atmospheric turbulence results in wavefront distortion, deviation in propagation direction, and oscillations in energy distribution by modifying the air’s refractive index [29,30]. Moreover, the intrinsic nonlinear drift in CMOS sensors leads to inaccuracies in slope calculations [31,32]. These elements collectively influence the system’s detecting precision.

**Figure 11:**
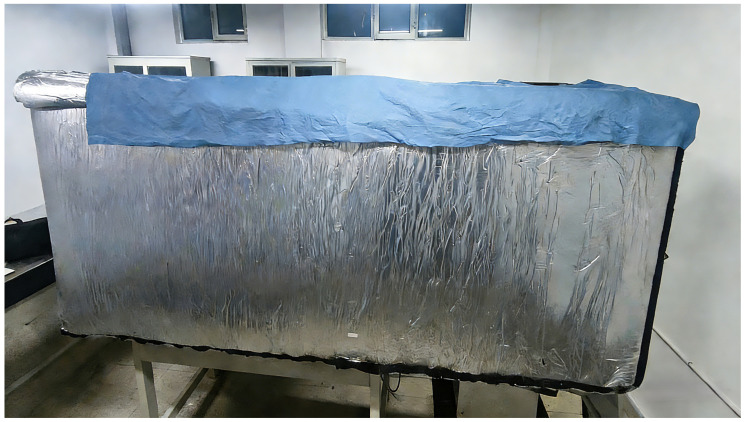
Shielding enclosure.

The experiment was conducted in a controlled indoor laboratory setting to reduce environmental interference and confine external influences. Nonetheless, indoor illumination and ambient sound produced persistent disruptions. To resolve this issue, we engineered and fabricated a 3 m long enclosed shielding structure, as depicted in Figure 11. The enclosure, fabricated from composite materials, effectively blocks external light when sealed. Internally, it is lined with sound-absorbing foam to attenuate both internal and external acoustic noise, while the enclosed volume minimizes disruptions from internal airflow.

**Figure 12:**
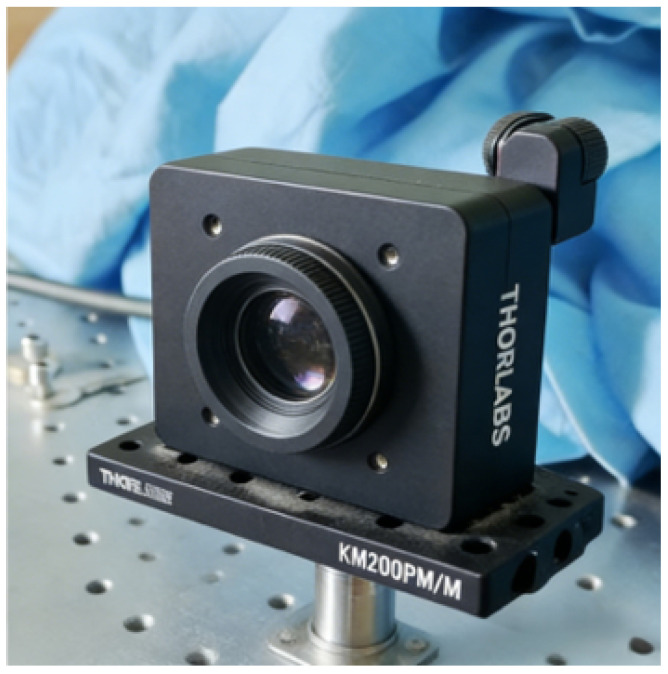
Shack–Hartmann wavefront sensor.

**Figure A1:**
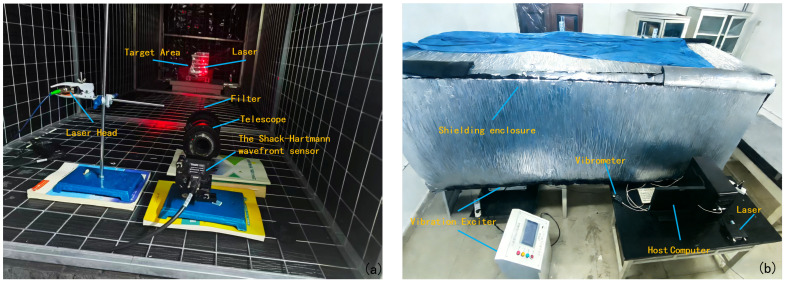
Diagram illustrating the collaborative interaction among system components. (**a**) Internal elements within the shielding enclosure; (**b**) External elements.

The Shack–Hartmann wavefront sensor (model WFS-20-5C, manufactured by Thorlabs, Newton, New Jersey, USA) was used to capture vibration signals, as shown in Figure 12. A 67 mW laser served as the source for detecting longitudinal vibrations originating from a target positioned 2 m from the sensor. For an overview of the system architecture and the operational interplay among its components, see Figure A1.

**Figure 13:**
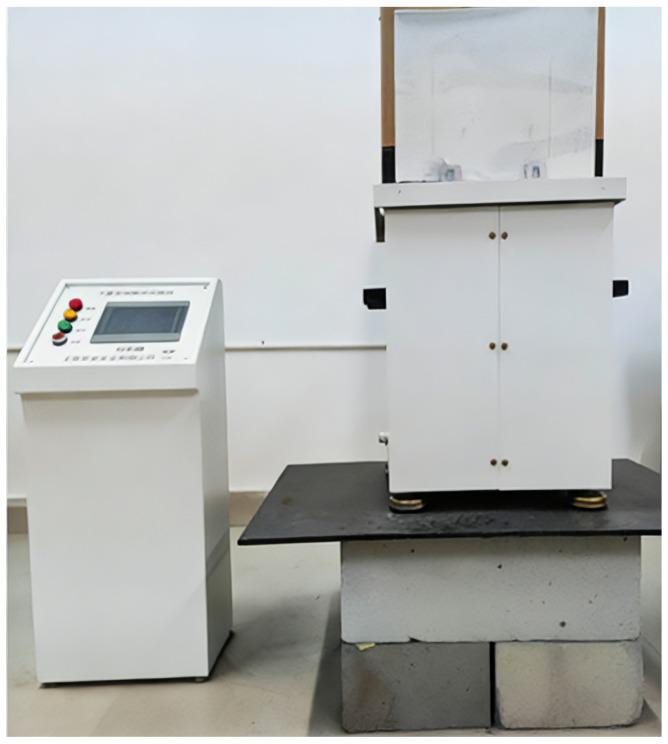
Controlled Vibration Platform.

The sensor incorporates automated shutter management, allowing it to adjust to a broad dynamic range of optical input power. A 635 nm wavelength laser was used as the light source due to the sensitivity of the wavefront sensor shown in Figure 5. To assess the ability of this wavefront sensor-based laser remote sensing system to detect vibration signals, a programmable vibration table was utilized as the vibration source, as seen in Figure 13. Vibration states with variable amplitudes were reproduced by altering the strength and frequency of the vibrations.

**Figure 14:**
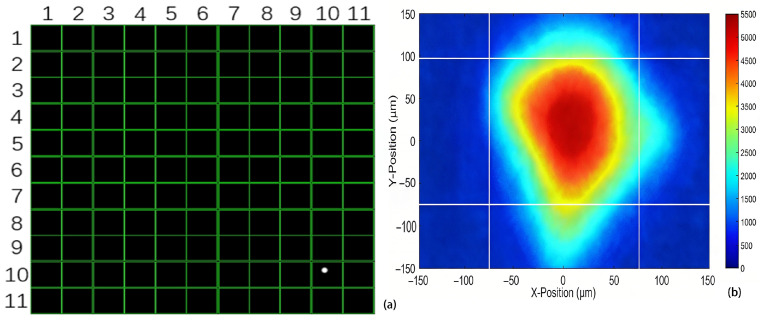
Partial reception locations within the microlens array. (**a**) A unique centroid location created by the laser reflection on the microlens; (**b**) signal points from the microlens at row 10, column 10 in the beam view mode.

During the experiments, the laser beam was directed at the vibration source, and the returning echo signals were collected. The wavefront sensor recorded vibration-induced spot centroid shift, from which the longitudinal vibration characteristics were analyzed. Data were acquired via the wavefront sensor software’s high-speed sampling mode, with the beam view interface displaying the spot distribution. Focusing the laser echo on the microlens at array position (row 10, column 10) produced a distinct centroid spot at that location (Figure 14a). Concurrently, signals of varying intensities appeared on adjacent microlenses (Figure 14b), indicating a “one-to-many” spatial coupling between the echo signal and the microlens array, as opposed to a strict one-to-one correspondence [11].

### 4.2. k Value Fitting in Vibration Detection

**Figure A2:**
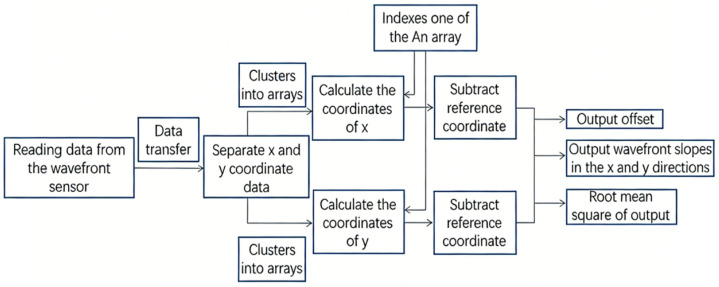
Calculation process.

We used LabVIEW software (version LabVIEW 2020.0 32-bit) for data collecting and analysis to ascertain the proportional link between vibration amplitude and spot centroid shift. The computational workflow is depicted in Figure A2. This software processes inputs from the wavefront sensor to compute the spot centroid shift at certain locations within the microlens array, specifically the 10th row and 10th column seen in Figure 14. This approach can be used for the entire 11 × 11 microlens array, enabling the centroid shift to be determined for all lenslets.

**Figure 15:**
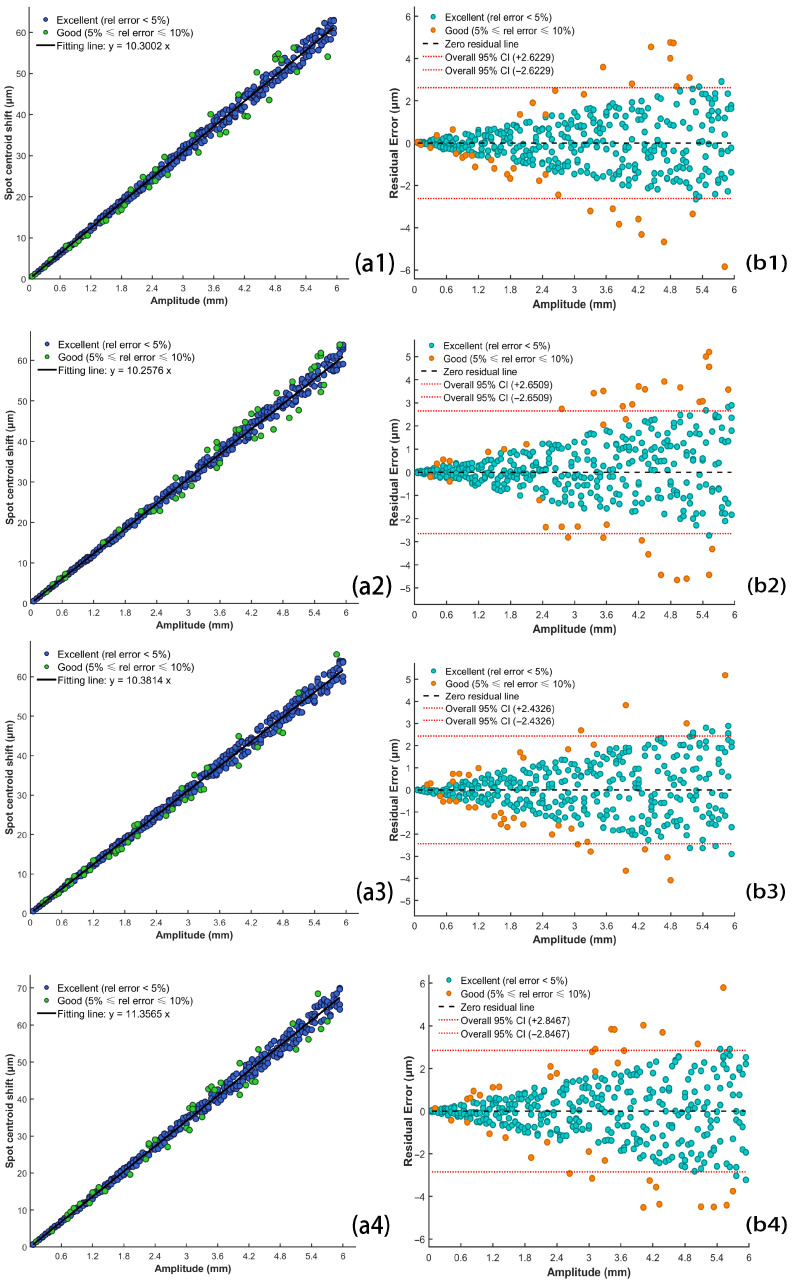
Correlation between vibration amplitude and spot centroid shift, including validation across several situations. (**a1**–**a4**) Fitted linear regressions of spot centroid shift against vibration amplitude; (**b1**–**b4**) residual distribution graphs.

Experiments revealed a positive correlation between vibration amplitude and spot centroid shift. To quantitatively assess this relationship, datasets were acquired over an amplitude range of 0.06–5.94 mm, and the slope *k* was determined via least-squares fitting. To further validate the robustness and generalizability of this correlation, repeated measurements and comparative analyses of the centroid shift were conducted under varying vibration frequencies, environmental conditions, and detection distances. The results are presented in Figure 15, while the corresponding experimental parameters are listed in Table 1.

Correlation error metrics were computed for the experimental setups illustrated in Figure 15. The results are summarized in Table 2.

**Figure A3:**
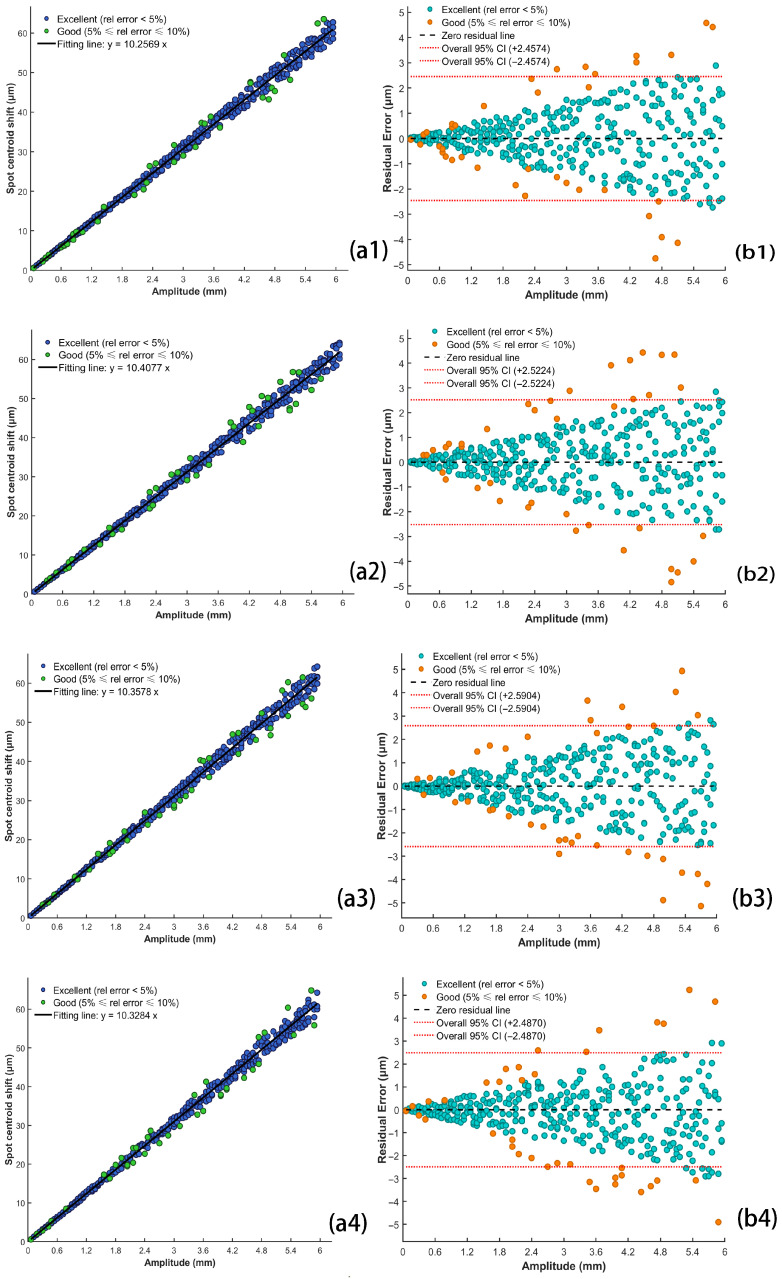
Correlation between vibration amplitude and spot centroid shift. (**a1**–**a4**) Fitted linear regressions of spot centroid shift against vibration amplitude; (**b1**–**b4**) residual distribution graphs.

To maintain the completeness and conciseness of the main text, detailed experimental configurations and supplementary test groups are presented in Figure A3, with the corresponding error metrics summarized in Table A2.

Within the shielding enclosure, varying the vibration frequency or the sensor receiver spacing yielded fitting coefficients ranging from 10.2576 to 10.3814, with the coefficient of determination $R^{2}$ exceeding 0.98. This indicates that, under controlled conditions, changes in frequency and sensor receiver spacing had minimal impact on the spot centroid shift, which remained within a stable range. The maximum value of the root mean square error (RMSE) reached 1.3609 μm, with the mean absolute error (MAE) hitting 0.9708 μm. In contrast, experiments conducted under natural environmental conditions revealed that the cumulative interference of ambient light and environmental noise significantly exacerbated the centroid shift. Compared to the shielding enclosure, the fitting coefficients increased markedly, and both RMSE and MAE were were elevated. Although the $R^{2}$ value could still reach 0.96, these fluctuations imposed a significant impact on the actual detection accuracy of the system.

### 4.3. Comparison of Vibration Velocity and Validation of Relationships

**Figure 16:**
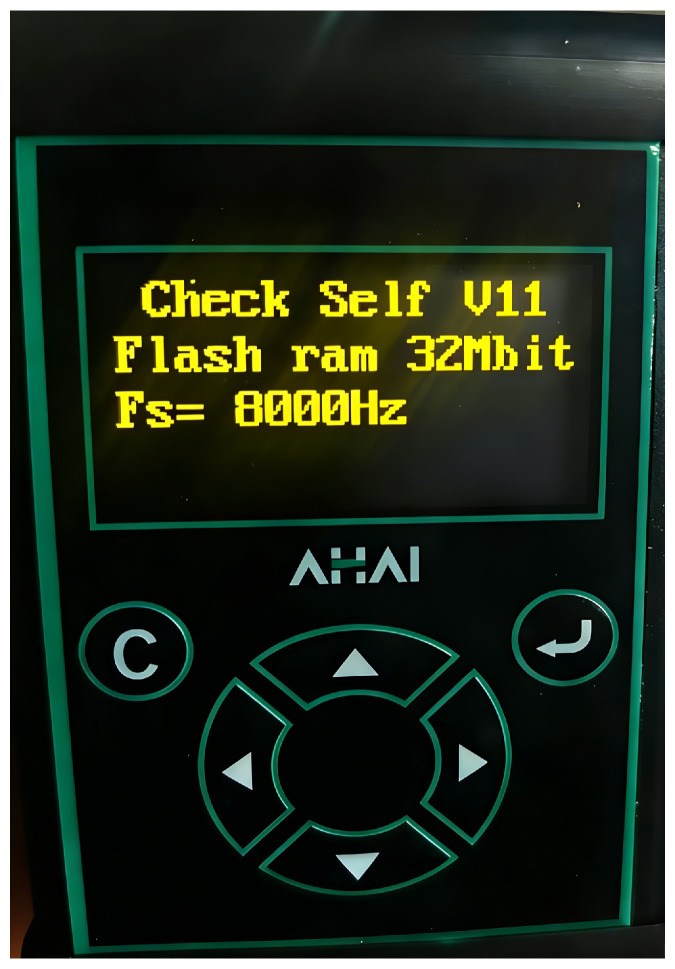
AHAI3001 Vibrometer.

Synchronous monitoring of the vibration table was conducted during the acquisition of vibration signals using the AHAI3001 Vibrometer(manufactured by Aihua Instruments Co., Ltd., Hangzhou, China), as shown in Figure 16. This instrument employs digital signal processing to simultaneously measure vibration velocity over specified time intervals. During acquisition, the AHAI Intelligent Host Management System software (version V1.7.0.0) displayed real-time vibration data from the device, with a sampling interval set to 10 ms.

**Figure 17:**
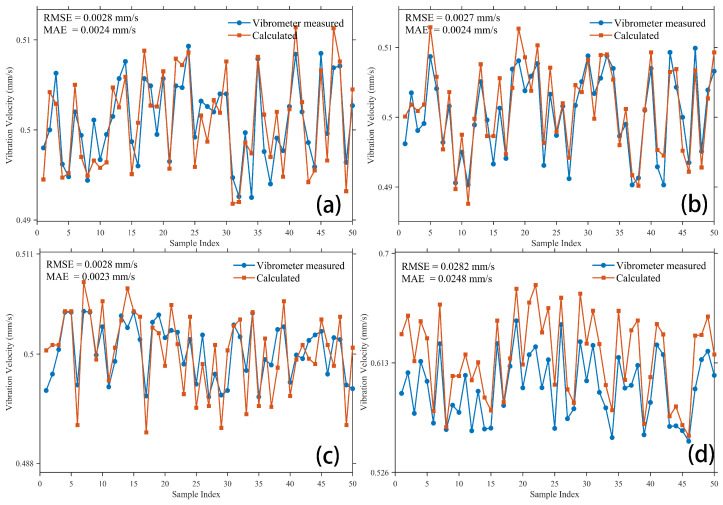
Comparative analysis of vibration velocity across varying situations (the experimental conditions listed in Panels (**a**–**d**) are frequency, amplitude, experimental environment, and sensor receiver spacing, respectively). (**a**) 0.1 Hz, 0.5 mm, shielding enclosure, 2 m; (**b**) 0.1 Hz, 0.5 mm, shielding enclosure, 1 m; (**c**) 1 Hz, 0.5 mm, shielding enclosure, 2 m; (**d**) 0.1 Hz, 0.5 mm, natural environment, 2 m.

**Figure A4:**
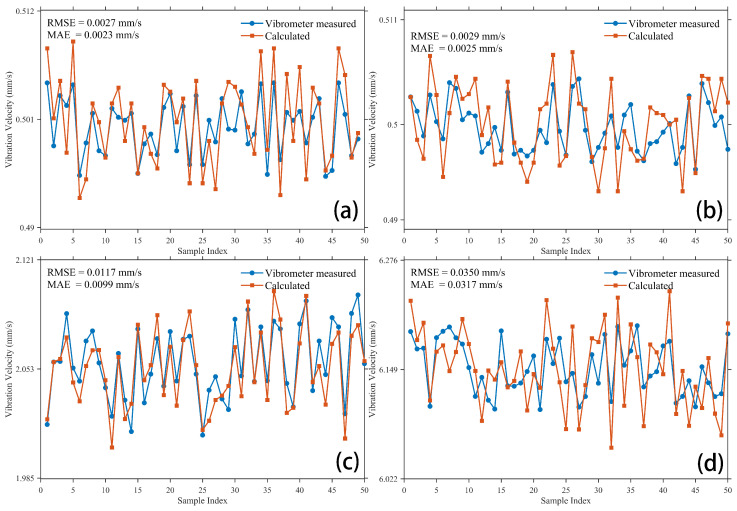
Comparative analysis of vibration velocity across varying situations (the experimental conditions listed in panels (**a**–**d**) are frequency, amplitude, experimental environment, and sensor receiver spacing, respectively). (**a**) 2 Hz, 0.5 mm, shielding enclosure, 2 m; (**b**) 0.1 Hz, 0.5 mm, shielding enclosure, 3 m; (**c**) 0.1 Hz, 2 mm, shielding enclosure, 2 m; (**d**) 0.1 Hz, 5 mm, shielding enclosure, 2 m.

To assess the reliability and generalizability of extracting vibration velocity from the spot centroid shift measured by the wavefront sensor, experiments were conducted under varying environmental conditions, vibration frequencies, and amplitudes. The vibration velocity derived from the centroid shift was compared with reference measurements obtained from a vibrometer. The comparison results are presented in Figure 17 and Figure A4.

The correlation error metrics for the experimental configurations presented in Figure 17 were quantified; the corresponding results are listed in Table 3.

Figure 17 presents a detailed comparison of experimental data obtained under diverse test conditions. Within the shielding enclosure, variations in frequency or sensor receiver spacing induced only minor fluctuations in vibration velocity, as evidenced by the low root mean square error (RMSE = 0.0027) and mean absolute error (MAE = 0.0023). In contrast, under natural environment, the vibration data measured by the vibrometer exhibited a slight increase, whereas the data extracted from the spot centroid shift displayed more significant variations. Correspondingly, the RMSE and MAE between the two datasets were 0.0282 and 0.0248, respectively.

Previous experimental results have demonstrated a linear relationship between vibration amplitude and the spot centroid shift measured by the wavefront sensor. Based on this observation, the value of vibration velocity derived from the spot centroid shift is expected to exhibit a corresponding linear relationship with vibration amplitude. To further examine the consistency of this relationship, additional analyses were conducted under the experimental conditions shown in Figure 15.

**Figure 18:**
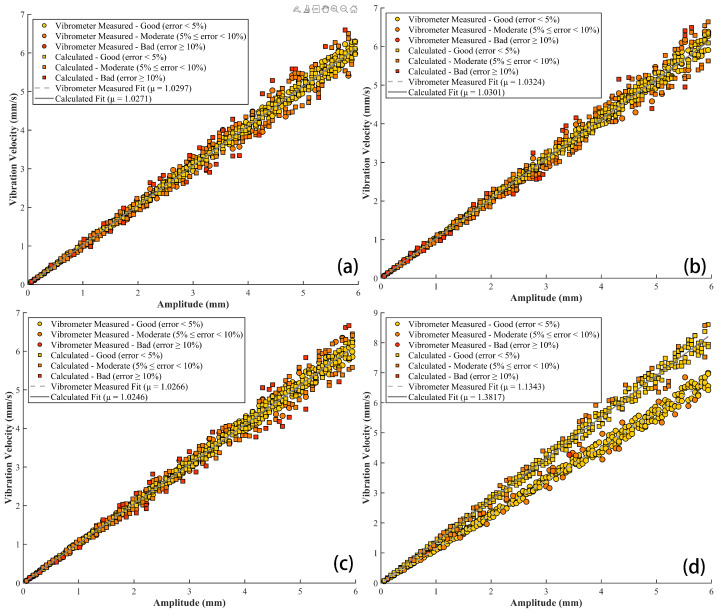
Relationship between vibration velocity and vibration amplitude under various experimental conditions (experimental conditions in (**a**–**d**) pertain to vibration frequency, sensor receiver spacing, and experimental environment, respectively). (**a**) 0.1 Hz, 2 m, shielding enclosure; (**b**) 1 Hz, 2 m, shielding enclosure; (**c**) 0.1 Hz, 3 m, shielding enclosure; (**d**) 0.1 Hz, 2 m, natural environment.

In these experiments, only a single variable was adjusted, with the vibration amplitude varying in the range of 0.06 mm to 5.94 mm. Figure 18 presents a comparison between the vibration velocity derived from the spot centroid shift and that measured by the vibrometer, along with the fitted relationship between vibration velocity and vibration amplitude.

**Figure A5:**
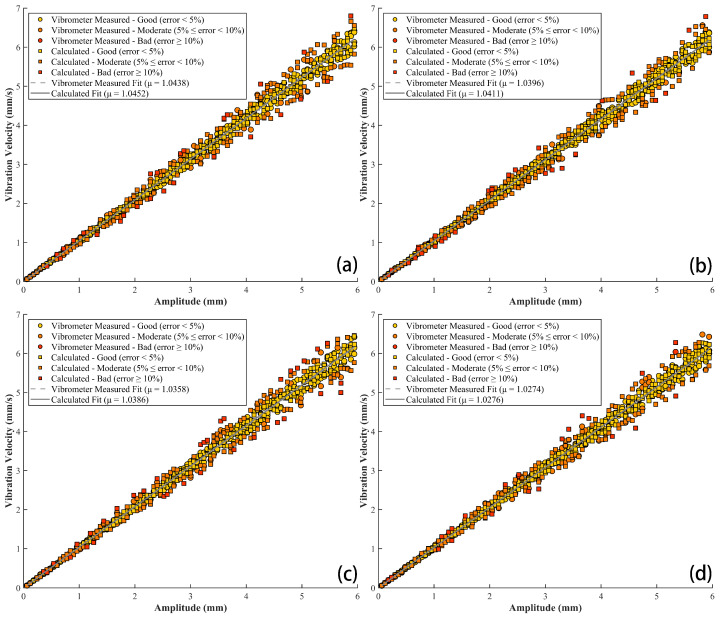
Relationship between vibration velocity and vibration amplitude under various experimental conditions (experimental conditions in (**a**–**d**) pertain to vibration frequency, sensor receiver spacing, and experimental environment, respectively). (**a**) 0.1 Hz, 1 m, shielding enclosure; (**b**) 0.2 Hz, 2 m, shielding enclosure; (**c**) 2 Hz, 2 m, shielding enclosure; (**d**) 5 Hz, 2 m, shielding enclosure.

Using the vibrometer measurements as the reference standard, we evaluated the consistency of the vibration velocity derived from the spot centroid shift under the experimental conditions shown in Figure 18 and Figure A5. The corresponding error metrics are summarized in Table 4 and Table A3. Additionally, the error metrics characterizing the linear relationship between the derived vibration velocity and vibration amplitude are presented in Table 5 and Table A4.

Analysis of the data presented in Figure 18 and Figure A5 indicates that, within the shielding enclosure, variations in either the vibration frequency or the distance between the sensor and the vibration source result in a fitted slope coefficient μ ranging from 1.0266 to 1.0438 when the vibrometer measured vibration velocity is regressed against vibration amplitude using the least-squares method. By comparison, the corresponding μ value obtained by fitting the vibration velocity derived from the spot centroid shift to the vibration amplitude range from 1.0246 to 1.0452.

As summarized in Table 4 and Table A3, the coefficient of determination $R^{2}$ between the vibration velocity derived from the centroid shift and the vibrometer measurements exceeds 0.98 in all cases, accompanied by a minimum RMSE of 0.1212, a minimum MAE of 0.1166, and a mean absolute percentage error (MAPE) below 5%. Furthermore, as shown in Table 5 and Table A4, the $R^{2}$ between the derived vibration velocity and its corresponding theoretical values obtained from the fitted regression line also exceed 0.97, with minimum RMSE and MAE of 0.1912 and 0.1355, respectively. These results demonstrate that, under controlled shielding enclosure conditions, the vibration velocity derived from the spot centroid shift exhibits strong agreement not only with vibrometer measurements but also with the theoretical values predicted by the linear model.

In contrast, under natural environmental conditions, measurement accuracy deteriorates substantially due to increased interference. The slope coefficient μ obtained by the vibrometer measured velocity against vibration amplitude increased to 1.1343. The corresponding correlation coefficient $R^{2}$ between vibrometer measurements and values derived from the spot centroid shift dropped to 0.7931, accompanied by an RMSE of 0.8860, an MAE of 0.7428, and a MAPE of 21.84%. Meanwhile, the μ value derived from fitting the spot centroid shift to the amplitude rose to 1.3817.

Although the derived vibration velocity under natural conditions maintained a high linear correlation with the theoretical regression values ($R^{2}$ = 0.9647), the associated error metrics—an RMSE of 0.2717 and an MAE of 0.2245—reveal notable discrepancies between model predictions and measured data. The above error metrics indicate that, in natural environments, the combined effects of various error factors substantially impact both the precision and accuracy of the system’s measurements.

## 5. Conclusions

This study presents a seismic laser remote sensing detection system that leverages the high precision, sensitivity, and array-based detection capabilities of a Shack–Hartmann wavefront sensor to acquire vibration signals. Through a series of experiments conducted under diverse and controlled conditions, the relationships among key quantities—including vibration amplitude, spot centroid shift, and vibration velocity—were systematically investigated. The results demonstrate the feasibility of utilizing spot centroid shift information for vibration signal characterization in seismic-related applications.

The fundamental configuration of the proposed detection system, along with the evolution of the optical wavefront under vibrational excitation, was first described. The wavelength response characteristics of the Shack–Hartmann wavefront sensor were analyzed, and a laser transmission attenuation model and the impact of temperature and humidity on wavefront sensor performance were established to examine the influence of laser output on signal acquisition. Experiments involving low-frequency, small-amplitude vibrations were then conducted. Spot centroid shift data were systematically collected under controlled vibration conditions with amplitudes ranging from 0.06 mm to 5.94 mm, from which vibration velocity were derived. The experimental results reveal a pronounced linear relationship between vibration amplitude and spot centroid shift, with coefficients of determination $R^{2}$ exceeding 0.98. A similarly strong linear relationship was observed between vibration amplitude and vibration velocity, with $R^{2}$ values consistently above 0.97. Moreover, vibration velocity derived from the wavefront sensor data close agreement with those measured by a reference vibrometer, indicating that the proposed approach is suitable for low-frequency vibration detection within controlled experimental environments.

Multiple experimental scenarios were designed to investigate the system’s response characteristics under varying conditions and to complete the acquisition and processing of vibration signals. Across these scenarios, the consistency between theoretical derivations based on spot centroid shift and measurements obtained from the vibrometer was systematically verified. These results provide robust experimental support for subsequent vibration parameter inversion studies for extending this laser remote sensing approach to broader-scale applications, including potential deployment on unmanned aerial vehicle (UAV) platforms for flexible and seismic monitoring.

It should be noted that the present experiments were primarily conducted using a vibration table under controlled laboratory conditions. The system’s performance under complex outdoor environments, including its resistance to environmental interference, has not yet been fully evaluated. Factors such as laser power attenuation during long-distance propagation, as well as the effects of temperature and humidity variations on detection stability, require further investigation. Future work will focus on field experiments to assess system robustness under realistic conditions. Potential improvements include the use of higher-power laser sources under favorable atmospheric conditions and the application of data-driven noise suppression techniques, such as neural network-based signal processing, to enhance signal-to-noise ratio. These efforts aim to support the eventual integration of the proposed system with UAV platforms for efficient, flexible, and wide-area seismic laser remote sensing applications.

## Figures and Tables

**Table 1:** Experimental parameters associated with each subfigure in Figure 15.

Subfigure ID	Frequency (Hz)	Experimental Environment	Sensor Receiver Spacing (m)	Fitted *k* Value
Figure 15(a1,b1)	0.1	Shielding enclosure	2	10.3002
Figure 15(a2,b2)	1	Shielding enclosure	2	10.2576
Figure 15(a3,b3)	0.1	Shielding enclosure	3	10.3814
Figure 15(a4,b4)	0.1	Natural environment	2	11.3565

**Table 2:** Assessment of linear fitting errors under varied experimental conditions.

Subfigure ID	$R^{2}$	RMSE (μm)	MAE (μm)	Fitted *k* Value
Figure 15(a1)	0.9843	1.3368	0.9708	10.3002
Figure 15(a2)	0.9841	1.3609	0.9498	10.2576
Figure 15(a3)	0.9952	1.2395	0.9425	10.3814
Figure 15(a4)	0.9644	1.5595	1.2634	11.3565

**Table 3:** Error metrics for the comparison of vibration velocity.

Subfigure ID	RMSE (mm/s)	MAE (mm/s)
Figure 17a	0.0028	0.0024
Figure 17b	0.0027	0.0024
Figure 17c	0.0028	0.0023
Figure 17d	0.0282	0.0248

**Table 4:** Error metrics for measured and derived vibration velocity under various conditions.

Subfigure ID	$R^{2}$	RMSE (mm/s)	MAE (mm/s)	MAPE (%)
Figure 18a	0.9826	0.1573	0.1166	3.98
Figure 18b	0.9821	0.1573	0.1203	4.02
Figure 18c	0.9820	0.1595	0.1209	4.01
Figure 18d	0.7931	0.8860	0.7428	21.84

**Table 5:** Error metrics for theoretical value and derived vibration velocity under various conditions.

Subfigure ID	$R^{2}$	RMSE (mm/s)	MAE (mm/s)	MaxAE (%)
Figure 18a	0.9767	0.2037	0.1450	16.66
Figure 18b	0.9772	0.2019	0.1434	16.12
Figure 18c	0.9882	0.1953	0.1443	17.53
Figure 18d	0.9647	0.2717	0.2245	9.84

## Data Availability

The data presented in this study are available on request from the corresponding author.

## Abbreviations

The following abbreviations are used in this manuscript:

CMOS	Complementary Metal Oxide Semiconductor
SHWFS	Shack–Hartmann wavefront sensor
RMSE	Root Mean Square Error
MAE	Mean Absolute Error
MaxRE	Maximum Relative Error
MAPE	Mean Absolute Percentage Error
RH	Relative Humidity
VIS	Visibility
UAV	Unmanned Aerial Vehicle

## Appendix B

First, the raw spot image data is read from the wavefront sensor. Subsequently, a clustering algorithm is applied to partition the spot pixels into several subregions, and the intensity distribution of each subregion is organized into a data array. For each subregion, the x- and y-coordinates of the spot centroid are calculated and then subtracted from the pre-calibrated reference coordinates to obtain the offset for that subregion. Based on these offsets, the local wavefront slopes in the x and y directions are derived. Finally, the slopes from all subregions are statistically analyzed to output the RMS value, which is used to evaluate the overall wavefront distortion. This process enables real-time computation from raw images to wavefront slopes, providing foundational data for subsequent wavefront reconstruction and vibration analysis.

## Appendix C

**Table A1:** Experimental parameters associated with each subfigure in Figure A3.

Subfigure ID	Frequency (Hz)	Experimental Environment	Sensor Receiver Spacing (m)	Fitted *k* Value
Figure A3(a1,b1)	0.1	Shielding enclosure	1	10.2569
Figure A3(a2,b2)	0.2	Shielding enclosure	2	10.4077
Figure A3(a3,b3)	2	Shielding enclosure	2	10.3578
Figure A3(a4,b4)	5	Shielding enclosure	2	10.3284

**Table A2:** Assessment of linear fitting errors under varied experimental conditions in Figure A3.

Subfigure ID	$R^{2}$	RMSE (μm)	MAE (μm)	Fitted *k* Value
Figure A3(a1)	0.9849	1.2507	0.9021	10.2569
Figure A3(a2)	0.9848	1.2896	0.9274	10.4077
Figure A3(a3)	0.9845	1.3199	0.9689	10.3578
Figure A3(a4)	0.9847	1.2751	0.9123	10.3284

## Appendix E

**Table A3:** Error metrics for measured and derived vibration velocity under various conditions.

Subfigure ID	$R^{2}$	RMSE (mm/s)	MAE (mm/s)	MAPE (%)
Figure A5a	0.9811	0.1656	0.1248	4.04
Figure A5b	0.9812	0.1530	0.1208	4.10
Figure A5c	0.9806	0.1660	0.1269	4.06
Figure A5d	0.9916	0.1212	0.1212	3.94

**Table A4:** Error metrics for theoretical value and derived vibration velocity under various conditions.

Subfigure ID	$R^{2}$	RMSE (mm/s)	MAE (mm/s)	MaxAE (%)
Figure A5a	0.9861	0.2087	0.1461	17.50
Figure A5b	0.9859	0.2134	0.1531	15.66
Figure A5c	0.9857	0.2142	0.1511	16.30
Figure A5d	0.9883	0.1912	0.1355	17.02
